# The Value of Providing Smokers with Free E-Cigarettes: Smoking Reduction and Cessation Associated with the Three-Month Provision to Smokers of a Refillable Tank-Style E-Cigarette

**DOI:** 10.3390/ijerph15091914

**Published:** 2018-09-03

**Authors:** Neil McKeganey, Joanna Astrid Miler, Farhana Haseen

**Affiliations:** Centre for Substance Use Research, Block 4, West of Scotland Science Park, Kelvin Campus, Block 302, Glasgow G20 OSP, UK; j.miler@csures.org (J.A.M.); haseen@csures.org (F.H.)

**Keywords:** e-cigarettes smoking cessation free provision

## Abstract

Despite the uptake of tobacco smoking declining in the United Kingdom (UK), smoking is still the leading cause of preventable poor health and premature death. While improved approaches to smoking cessation are necessary, encouraging and assisting smokers to switch by using substantially less toxic non-tobacco nicotine products may be a possible option. To date, few studies have investigated the rates of smoking cessation and smoking reduction that are associated with the provision of free electronic-cigarettes (e-cigarettes) to smokers. In this exploratory study, the Blu Pro e-cigarette was given to a convenience sample of adult smokers (*n* = 72) to assist them in reducing and quitting over a 90-day period. The rates of smoking abstinence and daily smoking patterns were assessed at baseline, 30 days, 60 days, and 90 days. The response rate was 87%. After 90 days, the complete abstinence rate was 36.5%, up from 0% at baseline. The frequency of daily smoking reduced from 88.7% to 17.5% (*p* < 0.001), and the median consumption of cigarettes/day reduced from 15 to five (*p* < 0.001). The median number of days per month that participants smoked also reduced from 30 to 13 after 90 days (*p* < 0.001). On the basis of these results, there may be value in smoking cessation services and other services ensuring that smokers are provided with e-cigarettes at zero or minimal costs for at least a short period of time.

## 1. Introduction

Despite rates of smoking uptake declining in the western world, tobacco smoking still continues to kill more people, cause more disease, and contribute more to social inequalities in high-income countries than any other preventable factor [1,2]. After stopping smoking, the associated health risks diminish substantially in proportion to the period of abstinence, with former smokers living longer than those who continue to smoke [3]. Quitting smoking at the soonest opportunity is the best action that a person can take to improve their health in the medium to long-term [3]. Given that most of the eight million smoking-related deaths that are projected to occur globally by 2030 will be among current, not prospective smokers [4], developing new effective ways of helping people quit smoking is a public health imperative.

According to Public Health England (PHE) and the Royal College of Physicians, electronic cigarettes (e-cigarettes) are likely to be at least 95% less harmful than smoking [1,5]. This statement has been recently affirmed, with PHE re-stating that e-cigarettes pose a fraction of the harms that smoking does, and that smokers should be encouraged to switch to e-cigarettes [5]. Whilst it is widely accepted that e-cigarettes are substantially less harmful than combustible cigarettes, nevertheless, these devices are not risk or harm-free. Along with the uncertainty regarding the health impact of long-term e-cigarette use, concerns have also been raised as to more immediate cardiovascular and other health harms that have been linked to e-cigarette use [6,7]. Although it is widely acknowledged that the complete cessation of all tobacco and nicotine use is the best action that smokers can take to improve their health, the Royal College of Physicians and Public Health England have both indicated the likely benefit of switching to the exclusive use of vaping devices for those smokers who are unable or unwilling to quit smoking combustible tobacco products. The issue that we address within this paper is whether the encouragement and assistance to smokers to switch to non-combustible tobacco products should include the free provision of those products for at least a restricted period of time.

### The Evidence Base on the Positive Role of E-Cigarettes in Smoking Cessation

A growing body of evidence is beginning to emerge that suggests that e-cigarettes can be an effective tool in helping people quit smoking [1,5]. Since 2013, e-cigarettes have been the most common quitting aid for smokers in England, according to Public Health England [5]. That finding has been affirmed in recent data showing that 38.2% of people in the last quarter of 2017 report using an e-cigarette in their recent quit attempt compared with 18% using nicotine replacement therapy (NRT) and 2.8% using varenicline (Champix) [8]. Regarding the efficacy and effectiveness of e-cigarettes in quitting smoking, the latest data suggests that tank models are superior to cig-alike e-cigarettes. Using a tank model may increase the odds of successfully quitting smoking by up to 2.19 times [8].

Studies investigating abstinence rates found that e-cigarettes are helpful in enabling smokers to switch. For example, the Eurobarometer 2017 study [9] of e-cigarette use in 28 Member States of the European Union utilised responses from 3612 participants (current or ex-smokers who at least tried e-cigarettes in the past), and found that 14% of respondents indicated that e-cigarettes had enabled them to stop smoking tobacco entirely. A recent study by Manzoli et al. [10] followed up 236 e-cigarette users (all of whom were ex-smokers), 491 smokers, and 232 dual users for 12 months, and found that 61.9% of the vapers were still abstinent from tobacco smoking, compared with just 20.6% of the smokers and 22.0% of the dual users, again suggesting that e-cigarettes can be effective in helping smokers abstain.

Data from the Eurobarometer 2017 study [8] indicated that 17% of the 3612 respondents reported a reduction in their tobacco consumption due to the use of e-cigarettes (but not complete cessation). Another study showed a reduction in cigarette smoking rates within a six-week period [11]. Our study adds to this literature by reporting both the rates of complete cessation as well as smoking reduction, which are associated with a 90-day ad libitum use of a tank-style e-cigarette in smokers.

## 2. Methods

As this is an exploratory trial, formal sample calculation was not undertaken. The study recruited 72 adult (18–65 years) smokers from Glasgow in the United Kingdom (UK) who indicated a willingness to try e-cigarettes as a method of quitting smoking. These respondents agreed to try using an e-cigarette in place of combustible cigarettes for 90 days.

Participants were recruited through a variety of means: an advertisement placed in a local Glasgow newspaper (*Glasgow Evening Times*); posts on social media accounts belonging to the investigators; local targeted (by postcode) leaflet drop (see Appendix A), including within areas with lower socio-economic status (which are known to have higher rates of smoking); leaflet distribution in Glasgow city centre; liaison with human resource departments of Glasgow-based companies; and word of mouth/snowballing. The particular brand name (Blu Pro) or device type was not indicated in advertisements for the research. Eligibility to participate was determined by the use of an initial screening questionnaire (see Appendix B). All of the participants in this study were provided with detailed information booklets outlining the study and describing in detail the e-cigarette that they would be using (Supplementary files 1 and 2).

Using these multiple methods, we recruited adults (aged 18–65) who had been smoking at least 10 cigarettes/day on the days they were smoking for at least the past 12 months, and who weren’t already regular e-cigarette users (maximum e-cigarette use in the past month was five occasions). Participants were required to not have used any NRT or stop smoking medication in the past 30 days and 90 days, respectively, nor to have had behavioral support to quit smoking in the past 30 days. Pregnant or lactating women were excluded. All of the potential participants were emailed the study information sheet.

Each eligible participant was invited to a face-to-face meeting with the lead researcher on this study (JM). At this meeting, individuals were shown the Blu Pro device, and offered the opportunity to choose from a range of flavors (tobacco, menthol, blueberry, cherry, and strawberry mint) and three nicotine strengths (tobacco—16 mg/mL, 8 mg/mL; menthol—16 mg/mL, 8 mg/mL; blueberry—16 mg/mL, 0 mg/mL, cherry—8 mg/mL, 0 mg/mL; and strawberry mint—8 mg/mL, 0 mg/mL), making 10 flavors and nicotine strength combinations. Disposable mouth tips were used in the training for the purpose of hygiene. At the conclusion of this meeting, participants were provided with three 10-mL bottles of their preferred combination(s) of e-liquid to begin the study. It was explained to respondents that they would be free to choose from the other flavors and nicotine strengths during the three-month period of the study, although it was explained that they should use only Blu flavors within the Blu device provided to them.

Participants received training on how to assemble, charge, fill, and puff on the e-cigarette, culminating in having to demonstrate competency and confidence to use the e-cigarette and e-liquids safely and effectively themselves by assembling and filling up the e-cigarette with the e-liquid of their choice, and puffing on it. The investigator alerted the participants to a list of potential health warnings associated with the use of this e-cigarette that was issued by the manufacturer, and described the recommended actions in the event of a technical malfunction or adverse health effects. Participants were encouraged to ask questions about the operation and proper use of the Blu Pro e-cigarette throughout this session. While no instruction was given as to how frequently or deeply to puff on the e-cigarette, practicing different puffing styles was encouraged to find the regimen resulting in the most satisfying draw of vapour. Participants were told to try to use their e-cigarette instead of smoking whenever they had a craving for nicotine or an urge to smoke.

Beyond an initial free supply of the Blu Pro Kit and three 10-mL e-liquid refill bottles, participants were told to purchase any additional refills from stockists in the community or from online vendors (reimbursed by cheque or bank transfer, up to the value of £30/month/participant (the cost of six refill bottles), upon presentation of valid receipts). In the event of a breakage or malfunction in the e-cigarette provided, the research team was able to ensure a rapid replacement or reimbursement where the study participant had purchased a new device. It was explained to study participants that the research team was unable to reimburse the use of other liquids than those manufactured for the Blu Pro device.

Participants completed an online questionnaire on four occasions—on day 1 (baseline), day 30, day 60, and day 90. On these days, they were to receive an email containing a web-link to an online questionnaire (hosted online on SurveyMethods) and were to complete the questionnaire within three days of receipt of the email. Participants were paid £50.00 as a compensation for their time in completing the survey instrument at each follow-up point.

### 2.1. Ethics Approval

The study design was reviewed by the West of Scotland Research Ethics Committee, which judged that the design of the study, including the use of commercially available e-cigarettes, meant that the study could proceed without a full committee assessment.

### 2.2. Measures

Participants were asked to report on the frequency and quantity of cigarette consumption in the past 30 days.

#### 2.2.1. Rate of Abstinence

At baseline, all of the participants were smokers. The “abstinence rate” was the percentage of those who did not smoke in the last 30 days at the time of the interviews (day 30, day 60, day 90) among those who were smoking before the interviews. Participants were asked, “In the past 30 days, have you smoked a cigarette, even one or two puffs?” Response categories were ‘Yes’ and ‘No’.

#### 2.2.2. Smoking Prevalence

Daily smoking prevalence was assessed by asking, “Do you now smoke cigarettes?” Possible responses were ‘Every day’, ‘Some days’, and ‘Not at all’. Participants who responded ‘Every day’ were coded as a ‘daily smoker’.

#### 2.2.3. Frequency and Amount of Smoking

Current users of cigarettes were asked to write the ‘number of days’ in response to the question, “On how many of the past 30 days did you smoke cigarettes?” For those days on which they indicated that they did smoke, respondents were asked to indicate how many cigarettes they typically smoked each day. The use of cigarettes per day was then categorised into three levels: light (1–10), moderate (11–20), and heavy (>20).

Baseline characteristics were collected within three days of the training session by the study investigator. Demographic data included age, gender, ethnicity, and socio-economic status. Questions relating to smoking history assessed the duration of cigarette smoking (years); the age of the first cigarette smoked (years); the age at which the individual started smoking daily (years); and previous quitting attempts.

Other measures relating to the frequency of use of the e-cigarette; perceived helpfulness; and the use of flavors were also collected.

### 2.3. Statistics

Given that this is a single arm trial, descriptive statistics were used to calculate the rate of abstinence, daily smoking prevalence, and frequency and amount of smoking. The primary analyses of outcomes included all of the enrolled participants who enrolled into the study and completed baseline, but follow-up was censored if a participant was lost to follow-up (day 30 *n* = 7, day 60 *n* = 5, and day 90 *n* = 9). An intent-to-treat (ITT) approach with imputations was not applied, since this is more appropriate for randomised control trials.

Bivariate associations of smoking behaviors between baseline and following initiating e-cigarette use were assessed with *z*-tests and χ^2^ tests for categorical variables. Since data did not follow normal distribution, a non-parametric test (*Wilcoxon signed rank test*) was used for continuous variables, and descriptive data were presented as a median. This exploratory analysis concluded not to proceed with advanced models adjusted for confounders. All of the data was analysed using SPSS Version 25.

## 3. Results

A total of 72 participants were enrolled into the study, out of which four were lost to follow-up after baseline, indicating a low attrition rate (5.6%). Out of 72, 71 completed the baseline; 63 participants (87.5%) completed four measures (baseline, 30 day, 60 day and 90 day). More than 90% completed 30-day (90.2%, 65/72) and 60-day surveys (93.0%, 67/72); 87.5% completed the 90-day survey. Missing responses for outcome measures across the follow-up periods varied from 6.9% to 12.5%.

The baseline socio-demographic characteristics and smoking behaviors of the participants are presented in Table 1. More than three-quarters (79%) of the participants were ≤45 years old (average 35.7 years), ranging from 19 to 59 years. There were more men than women in the study (63.9% versus 36.1%). Most of the participants were British/Irish white (94.5%), and around three-quarters were single (70.8%). More than half were in full-time employment (56%) and earned less than £20,000 per year (62.5%). Participants varied in their levels of education, from no qualifications (11.3%) to Masters-level degree (2.8%), with the highest frequencies being those who obtained either an undergraduate university degree (22.5%) or a higher national certificate/diploma (25.4%).

The average age of those participants who were lost follow-up was 46 years (SD = 8.6, range 41–56 years); three were female and two were married and not currently unemployed. Due to the small numbers, it was not possible to test whether the loss of follow-up participants was different from those participants who completed the survey.

The age at which participants first tried a cigarette (even a single puff) ranged from seven to 25 years, with an average age of 14.3, and the age at which participants started smoking regularly or daily ranged from 11 to 26 years old, with an average age of 16. Participants varied in the length of time that they had been smoking, from just under one year to over 44 years; the majority of the sample were daily smokers (88.7%). Around two-thirds of participants were moderate to heavy smokers (62.5%), and their consumption of cigarettes/day varied from two to 30, with the median number of cigarettes being 15/day. More than half of the participants had tried to quit smoking in the past year (52.1%), and a majority of the participants indicated that they planned to quit smoking for good at some point in the future (95.8%). Over half (55.3%) wanted to quit in the next seven days, and 5.3% indicated their desire to quit in a year’s time.

### 3.1. Abstinence Rates after 90 Days of Ad Libitum Use of an E-Cigarette

**Figure 1:**
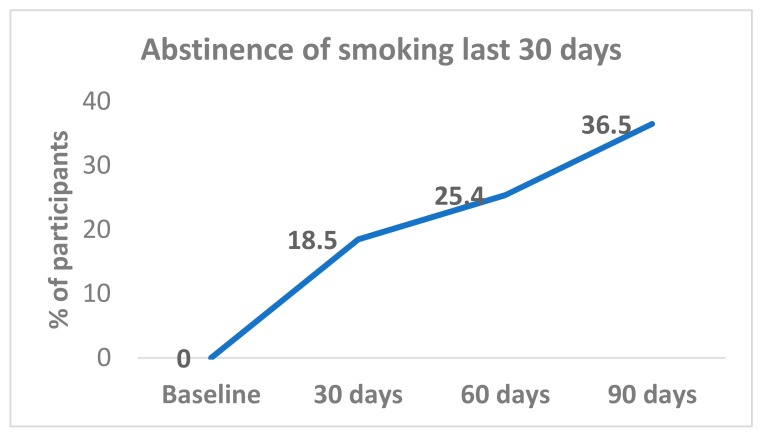
Past 30 days smoking abstinence levels.

All (100%) participants were smoking at baseline. Complete smoking abstinence was achieved by 18.5% (12/65) at day 30; 25.4% (17/67) at day 60; and 36.5% (23/63) at day 90 (Figure 1). Abstinence rates from day 30 were sustained with 72.7% (8/11) participants at day 60, and 81.8% (9/11) at day 90. Among participants who reported smoking abstinence at day 60, 87.5% (14/16) continued to be abstinent at day 90.

The changes in abstinence levels at the three different time points from baseline to 90 days were tested. The abstinence rate increased from baseline to the day-30 point and continued until the end of the study at 90 days, with significant differences between baseline and the three follow-up points (day 30, day 60, and day 90); *p* < 0.001. However, in relation to the time between follow-up points, the difference was statistically significant between day 30 and day 90 (18.5% versus 36.5%, *p* = 0.02); but not for day 30 versus day 60 or day 60 versus day 90.

### 3.2. Prevalence of Daily Smoking

**Figure 2:**
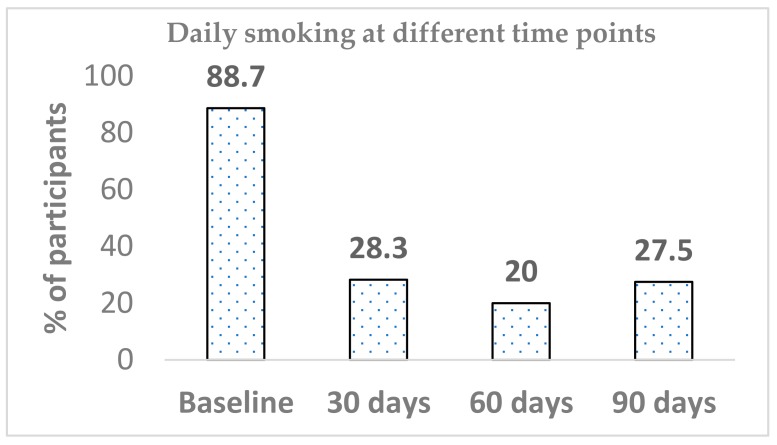
Prevalence of daily smoking.

A total of 22 participants did not respond to the daily smoking question on four occasions (baseline *n* = 1, day 30 *n* = 7, day 60 *n* = 5 and day 90 *n* = 9). Out of 71 participants, 63 at baseline reported smoking daily (88.7%), whilst the prevalence of daily smoking significantly dropped within 30 days of Blu Pro use (28.3%) (*p* < 0.001). Differences in daily smoking prevalence from between baseline to both day 60 and day 90 were significant (*p* < 0.001). The declining trend continued until the 60-day mark; then, there was an increase from 20.0% to 27.5%. The difference in daily smoking between day 60 and day 90 was not significant. Similarly, there was no further significant change in daily smoking between any other time points (day 30 versus day 60 or day 30 versus day-90) (Figure 2).

### 3.3. Cigarettes per Day

Participants ranged in their daily cigarette consumption from 2–30 cigarettes per day at baseline, 1–20 cigarettes per day at day 30, 1–25 cigarettes per day at day 60, and 1–20 cigarettes per day at day 90 (Table 2). The median number of cigarettes smoked per day dropped from 15 to three at day 30, four at day 60, and five at day 90; the differences was significant at all three time points from baseline (*p* < 0.001), but not between follow-up time points: day 30 and day 60, day 30 and day 90, and day 60 and day 90. A similar trend was observed when the number of cigarettes was categorised into three groups: low, moderate, and high. The low number of cigarettes that was consumed significantly increased from 36.6% at baseline to 89.2% just after starting Blu Pro for 30 days, and the low number of cigarettes consumption continued to drop for next two months, to 84.2% and 82.8% respectively. These differences were significant between baseline and all further time points (*p* < 0.001).

### 3.4. Number of Smoking Days

Participants who did not reach smoking abstinence ranged in their cigarette smoking between 3–30 days/month at baseline, and 1–30 days/month at day 30, day 60, and day 90 (Table 2). The baseline median number of days in the month that participants smoked (median 30) was significantly higher than at day 30 (median 15), day 60 (median 10), and day 90 (median 13) of the Blu Pro use (*p* < 0.001). Consistent with other outcome measures, these differences did not exist between follow-up time periods: day 30 versus day 60, day 30 versus day 90, and day 60 versus day 90.

### 3.5. Use and Acceptance of E-Cigarette

The Blu Pro was judged to be acceptable by study participants, almost all of whom (98.5%) used Blu Pro in the first 30 days; 95.5% continued to use the Blu Pro device for up to 60 days, and 81.0% continued using the Blu Pro for the entire 90 days. Out of the 12 participants who stopped using Blu Pro at day 90, eight (66.7%) neither smoked nor used any other e-cigarette. Overall, less than 10% reported the use of other e-cigarette brands at the 90-day follow-up.

Non-tobacco flavors were more popular than the exclusive tobacco flavor (56.9% versus 7.7% at day 30, 66.2% versus 15.4% at day 60, and 75.9% versus 13.8% at day 90; *p* = 0.013). The use of tobacco flavor significantly dropped from day 30 to day 90 (43.1% to 22.2%, *p* = 0.043). At every data collection point, the majority of smoking abstainers used non-tobacco flavors; among the 20 abstainers at day 90 (data available), two (10.0%) used the tobacco flavor, whilst the remaining 18 participants (90%) used flavored non-tobacco e-liquids.

All of the participants found that the flavors that were used were important in helping them quit or cut down, and 92.1% believed that the Blu Pro had helped them cut down or quit smoking at 90 days. Different nicotine strengths or flavors did not show either a trend or an association with smoking abstinence over time.

Overall, around three-quarters of participants (45/62; 73.8%) reported an intention to use the Blu Pro e-cigarette for the next month at the end of the study. Among the 22 abstainers, 15 (68.2%) expressed a plan to continue the Blu Pro in next month.

Self-reported purchases of liquid refills increased over time: 61% at day 30, 75% at day 60, and 77% at day 90; 10–25% participants bought >6 refills, which cost more than the reimbursement amount (10% at day 30, 25% at day 60, and 22%% at day 90).

## 4. Discussion

In this exploratory study, we have shown that the ad libitum use of e-cigarettes supported smokers’ efforts in quitting and reducing smoking over a 90-day period. The abstinence levels increased from baseline to day 30, and continued to rise throughout the study duration (90 days). The finding suggests that the use of vaping may have additional benefits with longer use—i.e., a proportion of smokers quit smoking within the first month of use, but a larger proportion needed more than two months to make the switch, and gradually quit over a longer period. After 90 days, we observed an abstinence rate of 36.5%. Other studies have reported rates of complete abstinence ranging from 14% [8] to 22.0% (of dual users at baseline) to 61.9% (in vapers at baseline after 12 months follow-up) [12]. Our results are not directly comparable; however, a 36.2% abstinence rate after three months is a promising result.

There are some limitations in our study. This was a small, exploratory, and uncontrolled study with a convenience sample and self-reported measures of smoking behavior. As a result, the findings should be interpreted with caution. Our research suggests that there may be benefits in providing smokers with access to free e-cigarettes and e-liquids for at least a short-term period. There can be little doubt as to the importance of encouraging and enabling smokers to cease smoking. Whilst the greatest health benefit can be achieved by smokers ceasing their smoking and nicotine consumption entirely, nevertheless, there are some smokers who wish to continue to use nicotine for at least a period of time. On the basis of this study, there is likely to be merit in providing smokers with access to e-cigarettes, letting them explore the various different flavors, and thereafter providing them with the opportunity to utilise these products either alongside or in the place of combustible products. As we saw even within a relatively short period of time, a significant number of smokers were able to cease smoking entirely over the study period, and others were able to reduce the numbers of days they smoked, as well as reduce the number of cigarettes per day smoked.

Whilst our study has shown that it is possible to facilitate significant behavioral change on the part of smokers as a result of providing them with access to alterative nicotine delivery systems (for at least a short period), an important question becomes one of whether these smokers were able to sustain those changed behaviors over an extended period of time. On the basis of our research, it was evident that notable changes in behavior were evident within the first month of being provided with the Blu Pro device and associated e-liquids, and that these were largely maintained throughout the three-month study period. Clearly, we cannot comment on the extent to which the positive reductions in smoking persisted beyond the three-month period over which respondents were provided with free products.

In the light of our positive results, there is a strong case for integrating the provision of alternative nicotine delivery systems within the existing stop smoking services, building upon the range of interventions that those services currently provide. Attention should be given to considering at what point in their contact with smokers service providers might consider providing free access to these devices. Clearly, such provision must not undermine smokers’ efforts to become completely abstinent. However, the inability of some smokers to quit smoking, coupled with the reality in which some smokers will choose to continue to smoke, even despite acknowledging the health harms associated with smoking, underlines why the simple “quit or die” message that is often conveyed to smokers could be usefully supplemented by the willingness of some smoking cessation services to provide smokers with access to these products at an appropriate point. Finally, it is important to acknowledge that even in the face of accepting the offer of free e-cigarettes and e-liquids over a three-month period, a notable minority of smokers in our study continued to smoke. Within the confines of the present study, it is not possible to determine whether those individuals would have reduced their smoking had we been offering them a greater range of vaping products. This is a possibility. It is also possible that some smokers will choose to continue to smoke, even in the face of the provision (at no cost) of alternative nicotine delivery systems. If this is indeed the case, it would suggest that e-cigarette manufacturers need to continue to innovate in product design and performance to attract new groups of smokers to use these devices as an alternative to combustible tobacco products. Similarly, there is a strong case in future research to assess the relative impact of different products in facilitating smokers switching from combustible to non-combustible tobacco products. Certainly, there is sufficient variation in the technology of alternative nicotine delivery systems to warrant research that can assess the speed with which different products facilitate that switch, and the extent to which different products are able to stimulate a sustained switch from smoking to exclusive vaping.

## 5. Conclusions

The reduction in smoking prevalence within the U.K. and in many countries over the last ten years has been one of the most notable achievements in public health promotion. However, even within those countries where smoking prevalence has dramatically reduced there is a continuing proportion of individuals who continue to smoke whilst acknowledging the harms and in the face of multiple measures aimed at discouraging their use of combustible tobacco products (bans on advertising, minimum age at which cigarettes can be legally purchased/used, plain packaging, in-door smoking bans, and price hikes through taxation). As a result there is a clear need to explore additional means by which more smokers might be encouraged to quit. The provision of e-cigarettes to smokers for at least a period of time, without financial charge, may well be something that services should consider. In the light of our findings there are clear indications that such an initiative might well have beneficial results in facilitating further quitting and further cessation in the use of combustible tobacco products.

## Figures and Tables

**Table 1:** Baseline Socio-Demographic Characteristics and Smoking Behaviors.

Variables	*n* = 72
Socio-demographic	
Age, mean (SD)	35.7 (11.4)
Gender, *n* (%)	
Male	46 (63.9)
Female	26 (36.1)
Marital status, *n* (%)	
Single	51 (70.8)
Married	14 (19.4)
Separated/divorced	6 (8.4)
Ethnicity, *n* (%)	
White: British/Irish	68 (94.5)
Other white	3 (4.2)
Employment, *n* (%)	
Part-time	7 (9.7)
Full-time	40 (55.6)
Unemployed	24 (33.3)
Yearly income, *n* (%)	
Between £0–20,000	45 (62.5)
Between £20,000–100,000	26 (36.2)
Smoking behaviors	
Age first tried smoking, mean (SD)	14.3 (2.7)
Age started smoking regularly, mean (SD)	16.3 (3.0)
Years being regular smoker, mean (SD)	18.0 (11.4)
Frequency of smoking, *n* (%)	
Daily	63 (87.5)
Some days	8 (11.1)
Cigarette per day, median (range)	15 (2–30)
1–10 (low)	26 (36.1)
11–20 (moderate)	39 (54.2)
>21 (high)	6 (8.3)
Attempt to quit in past 12 months	
Yes	37 (51.3)
No	34 (47.2)
Intention to quit smoking	
Yes	68 (94.4)
No	3 (4.2)

**Table 2:** Cigarette Use Per Day.

Cigarette Use	Baseline (*n* = 71)	30 Days (*n* = 37)	60 Days (*n* = 38)	90 Days (*n* = 29)	*p* Value *
Number of cigarettes/day median (range)	15 (2–30)	3 (1–20)	4 (1–25)	5 (1–20)	<0.001
1–10 (low), *n* (%)	26 (36.6)	33 (89.2)	32 (84.2)	24 (82.8)	<0.001
11–20 (moderate), *n* (%)	39 (54.9)	4 (10.8)	5 (13.2)	5 (17.2)	
≥21 (high), *n* (%)	6 (8.5)	0 (0.0)	1 (2.6)	0 (0.0)	
	*n* = 71	*n* = 53	*n* = 50	*n* = 40	
Number of days smoked median (range)	30 (3–30)	15 (1–30)	10 (1–30)	13 (1–30)	<0.001

* Significant from baseline using Kruskal–Wallis H (continuous variables) and chi-square (categorical).

## Supplementary Materials

The following are available online at http://www.mdpi.com/1660-4601/15/9/1914/s1, Supplementary file 1: Study information, Supplementary file 2: Blu Pro Kit Information.

## Appendix A

This leaflet was distributed as an aid to study recruitment. The reference to e-cigarettes as being 95% less harmful than smoking cigarettes was taken from the Public Health England evidence review report (2015). The heading on this leaflet “Time to Switch” was drawn from a similarly titled campaign run by the Leicestershire (UK) NHS Stop Smoking Service.

## Appendix B. Eligibility Screening Questions

1. Are you aged over 18?
2. Are you currently smoking?
3. Have you smoked at least 100 cigarettes in your lifetime and over the last 12 months have you smoked at least 10 cigarettes per day on the days that you have been smoking?
4. Have you ever used e-cigarettes?
5. Have you used an e-cigarette for the last 30 days?
6. Has a doctor or other health professional told you that you have any of the following conditions:
  - High blood pressure
  - High cholesterol
  - Congestive Heart Failure
  - Stroke
  - Heart Attack
  - Some other heart condition?
7. Has a doctor or other health professional told you that you have had:
  - COPD
  - Chronic bronchitis
  - Emphysema
  - Asthma
  - Some other respiratory condition?
8. Have you had a mental health problem diagnosed by a doctor or other health professional?
9. Have you ever been told that you have any type of cancer?
10. Are you pregnant, lactating?
11. Are you willing to try e-cigarettes as a possible aid to quitting smoking or reducing smoking?
12. Are you willing to complete a series of online questionnaires?
13. Are you related to somebody who is already participating in this study?
14. Does a family member or close friend work for the company manufacturing Blu e-cigarettes?
