# Supplementary material for: The Value of Providing Smokers with Free E-Cigarettes: Smoking Reduction and Cessation Associated with the Three-Month Provision to Smokers of a Refillable Tank-Style E-Cigarette
Source: Int J Environ Res Public Health. 2018 Sep 3;15(9):1914. doi: 10.3390/ijerph15091914 (PMC6165311; doi:10.3390/ijerph15091914)
Supplement: Supplementary file 1 [file ijerph-15-01914-s001.zip › Supplementary File 1. Study Information.pdf]

CSUR Research & Consultancy

Study Information Booklet

(Switchers Version)

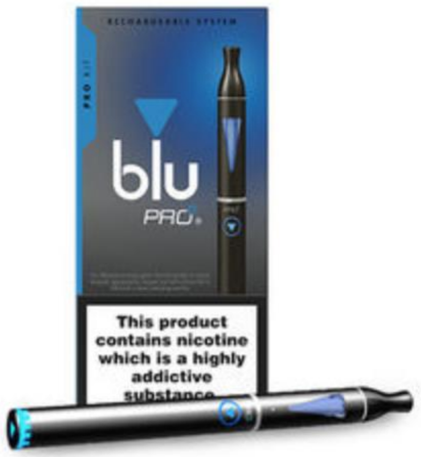

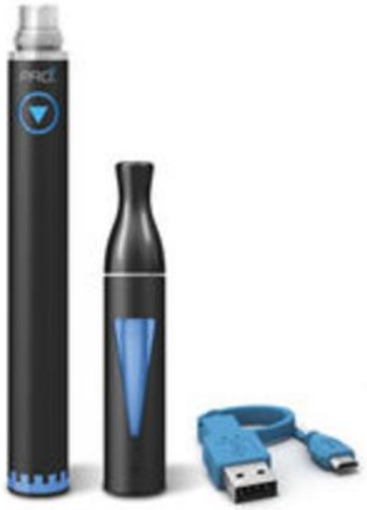

This Study Information Booklet is solely for use by individuals eligible to participate as 'Switchers' in the following study:

The effect of using the Blu PRO e-cigarette in place of smoking conventional cigarettes for 90 days.

Table of Contents

1 Why have I been given this Study Information Booklet? 4

2 Study title 4

3 Who are the Study Investigators? 4

3.1 Principal investigator 4

3.2 Supporting investigators 5

4 What is the purpose of this study? 5

5 Why have I been invited to take part? 5

6 What will I be required to do if I take part? 6

7 What are electronic cigarettes and how do they differ from tobacco products? ... 6

8 How safe are e-cigarettes? 7

9 Is there a risk to bystanders from electronic cigarette vapour? 8

10 What is the Blu PRO e-cigarette? 9

10.1 Understanding nicotine strengths 11

11 What happens if I choose to participate? 11

12 What happens next? 11

12.1 Learning how to use the Blu PRO e-cigarette 11

12.2 Vaping the Blu PRO for the first time 12

12.3 Finding your favourite flavour and nicotine strength of Blu Liquid 12

12.4 Using the Blu PRO and purchasing Blu Liquid refills between day-1 and day-90 13

13 Completing online questionnaires on day-30, day-60 and day-90 14

14 What are the possible risks of taking part? 15

14.1 Risk of harm from e-cigarette use 15

14.2 Risks associated with nicotine use 15

14.3 Risks associated with using the Blu PRO e-cigarette and e-liquids: 16

15 What are the potential benefits of taking part? 16

16 Will my identity and data be kept confidential? 18

17 Compensation for taking part and reimbursements 19

18 Who funded this research study? 19

19 What if I change my mind? 19

20 How can I find out more about this study? 19

21 I've decided I want to take part. What happens next? 20

22 I've decided I don't want to take part. What happens next? 20

1 Why have I been given this Study Information Booklet?

You are being invited to take part in a research study. It is important for you to understand why the research is being done and what it will involve prior to consenting to participate in the research. Please take time to read the information in this booklet carefully before deciding whether you wish to take part in this study. Take as much time as you need to come to a decision about whether you wish to take part in this study. You are free to take this booklet home to think about your decision, consider all the risks and benefits of participating, and seek advice from your friends and family. If you decide to take part, it is important that you are aware that you are still free to withdraw from the study at any stage without giving a reason.

2 Study title

The title of this study is: *The effect of using the Blu PRO e-cigarette in place of smoking conventional cigarettes for 90 days*.

3 Who are the Study Investigators?

The names and contact details of the four Study Investigators who will be overseeing every aspect of this study are provided below. You should keep these details to hand and feel free to contact any of these individuals at any time to ask any questions you may have about the study.

3.1 Principal investigator

Professor Neil McKeganey (Ph.D.)
Director
Centre for Substance Use Research
3.2 West of Scotland Science Park
Kelvin Walkway
Glasgow
G20 0SP
United Kingdom

P: 0141 9466392
E: mckeganey@csures.org

3.2 Supporting investigators

Dr Christopher Russell (Ph.D.)
Senior Research Fellow
Centre for Substance Use Research
3.2 West of Scotland Science Park
Kelvin Walkway
Glasgow
G20 0SP
United Kingdom
P: 07955460277
E: russell@csures.org

Dr Joanna Miler (Ph.D.)
Research Fellow
Centre for Substance Use Research
3.2 West of Scotland Science Park
Kelvin Walkway
Glasgow
G20 0SP
United Kingdom
P: 0141 9466392
E: j.miler@csures.org

4 What is the purpose of this study?

The main purposes of this study are:

1. To assess the ability and willingness of smokers to use a specific brand of e-cigarette – the Blu PRO – in place of smoking cigarettes for 90 days,

5 Why have I been invited to take part?

You have been invited to take part in this study because you are an adult smoker who has expressed an interest in using e-cigarettes as an alternative to smoking cigarettes.

You have also been invited to take part because you satisfy all the criteria required to be eligible to take part in this study.

6 What will I be required to do if I take part?

You are being asked to try to use a specific e-cigarette – the Blu PRO – in place of smoking regular cigarettes for the next 90 days. Specifically, whenever you feel a craving for nicotine or an urge to smoke a cigarette, we are asking you to vape the Blu PRO e-cigarette instead of lighting up a cigarette.

While we do want you to try your best to quit smoking cigarettes over the next 90 days, please note that you will not be excluded from this study if you do smoke any cigarettes at any time in the 90 days. That is, we are asking you to try to quit smoking, but we are not prohibiting you from smoking. We do, however, require you to be honest with us about when you smoke cigarettes and how many cigarettes you smoke, and likewise, to be honest about how often you use the Blu PRO e-cigarette. As such, a requirement for participation in this study is a commitment to complete a 5-minute online questionnaire about your cigarette and e-cigarette consumption on three occasions – on day-30, day-60 and day-90.

To clarify, we want you to try your best to use the Blu PRO e-cigarette in place of smoking regular cigarettes for 90 days, but you will not be excluded from the study if you do smoke any regular cigarettes.

7 What are electronic cigarettes and how do they differ from tobacco products?

The information below is found at the website of Action on Smoking and Health (http://ash.org.uk/stopping-smoking/ash-briefing-on-electronic-cigarettes-2/):

Electronic cigarettes, also known as vapourisers or electronic nicotine delivery systems (ENDS), are battery-powered devices that deliver nicotine by heating a solution of nicotine, flavouring, additives and propylene glycol and/or vegetable glycerine (glycerol). The devices typically consist of a mouthpiece, battery and cartridge or tank containing the nicotine solution.

When a user sucks on the device, a sensor detects air ow which activates a heating element (the ‘atomiser’) which heats the liquid in the cartridge so that it evaporates. The vapour delivers the

nicotine to the user. Electronic cigarettes were developed to mimic the action of smoking, including nicotine delivery, without the toxic effect of tobacco smoke.

When a person smokes a conventional tobacco cigarette, smoke is inhaled into the lungs and then exhaled. Smoke is also emitted from the burning tip of the cigarette, releasing toxins into the air. By contrast, as there is no combustion involved in the use of electronic cigarettes there is no smoke. Vapour is released into the air only when the user exhales.

There are three main types of electronic cigarettes or vapourisers:

1. “Cig-a-like” products: This first generation of electronic cigarettes were designed to resemble tobacco cigarettes. They sometimes have a light at the end that glows when the user draws on the device to resemble a lit cigarette. These consist of either non-rechargeable disposable models or an electronic cigarette kit that is rechargeable and includes replaceable pre-filled cartridges.

2. ‘Tank’ models (also known as vape pens): An electronic cigarette that is rechargeable and has a tank or reservoir which has to be filled with liquid nicotine. Tank models have now become more commonplace and allow the user to choose from a broader range of nicotine strengths and flavourings.

3. ‘Mods’ (or advanced personal vaporisers): A more complex tank model which can be manually customised by, for example, adjusting the voltage on the device.

8 How safe are e-cigarettes?

A report on e-cigarettes commissioned by Public Health England and published in 2015 concluded that, “In a nutshell, best estimates show e-cigarettes are 95% less harmful to your health than normal cigarettes, and when supported by a smoking cessation service, help most smokers to quit tobacco altogether.”

In a report published in 2016, the Royal College of Physicians concluded that “there appear to be few, if any, significant short-term adverse effects of e-cigarette use, but adverse health effects from long-term exposure to constituents of vapour can be ruled out. Although unknown, the hazard to health arising from long-term vapour inhalation is unlikely to exceed 5% of the harm from tobacco smoke. Switching from tobacco to e-cigarettes is therefore likely to be almost as effective in preventing harm as switching to NRT (nicotine replacement therapy)”.

This conclusion is consistent with a previous publication in which the Royal College of Physicians concluded that “as most of the harm caused by smoking arises not from nicotine but from other components of tobacco smoke, the health and life expectancy of today’s smokers could be radically improved by encouraging as many as possible to switch to a smoke-free source of nicotine.”

In line with these assessments of the safety of e-cigarettes relative to regular cigarettes, several health charities and organisations that function in large part to prevent and treat harm caused by smoking have united in agreement that promoting e-cigarettes, nicotine replacement therapies, and other non-tobacco nicotine products as widely as possible to smokers as a substitute for smoking has the potential to save the lives of millions of smokers in the United Kingdom. In particular, Cancer Research UK, the British Lung Foundation, the Royal College of General Practitioners, the Royal College of Midwives, and Action on Smoking and Health (ASH) have all signed a statement of agreement that e-cigarettes are significantly less harmful than regular cigarettes, and that smokers should be encouraged and supported to switch to e-cigarettes.

9 Is there a risk to bystanders from electronic cigarette vapour?

Most second-hand smoke from cigarettes comes from the burning tip, known as sidestream smoke. By contrast, electronic cigarettes do not generate any sidestream vapour. What is emitted into the air is exhaled by the electronic cigarette user. This comprises nicotine and some other particles, primarily consisting of flavours, aroma transporters, glycerol and propylene glycol. A recent review of the impact of electronic cigarettes found that passive exposure to the aerosol can expose non-users to nicotine but at concentrations that are unlikely

to have any significant health impact.

A 2015 report by Public Health England concluded that the amount of nicotine released into the ambient air poses no identifiable risk to bystanders. While electronic cigarette vapour can contain some of the toxicants present in tobacco smoke, these toxicants are founds at much lower levels in e-cigarette vapour. In 2016, the Royal College of Physicians reported that “there is, so far, no direct evidence that such passive exposure (to nicotine) is likely to cause significant harm…Nicotine from exhaled vapour can be deposited on surfaces, but at such low levels that there is no plausible mechanism by which such deposits could enter the body at doses that would cause physical harm”.

10 What is the Blu PRO e-cigarette?

If you choose to take part in this study, you will be provided with a Blu PRO e-cigarette, shown in Figure 1 below, free of charge. The Blu PRO is an open-system ‘tank-style’ electronic cigarette, which means it is rechargeable and has a tank/reservoir that is to be refilled with e-liquid. The Blu PRO e-cigarette is to be refilled with e-liquids manufactured by Blu, called ‘Blu Liquids’. Blu Liquids are sold in 10ml plastic bottles, and contain vegetable glycerine, propylene glycol, flavourings, nicotine and water. Blu Liquids are available for retail purchase in the United Kingdom, to individuals aged 18 years and older, in nine combinations of five flavours and three nicotine strengths, shown in Table 1 below. Images of the packaging of the five flavours of Blu Liquid are shown in Figure 2 below.

Figure 1. The Blu PRO Kit.

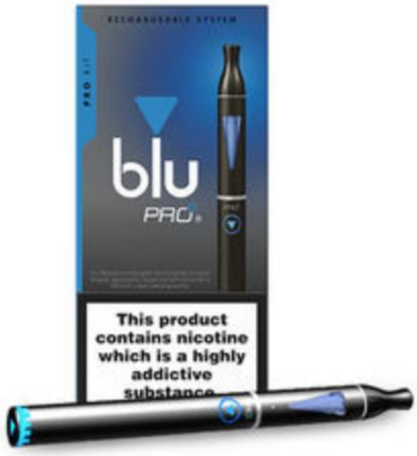

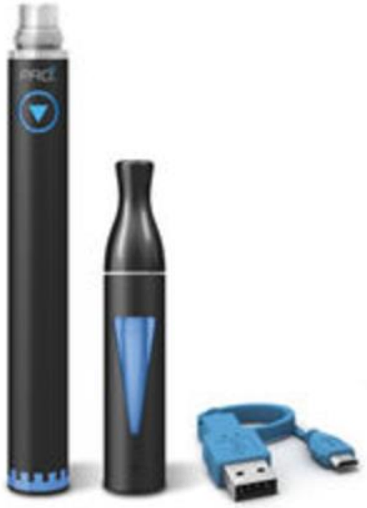

Table 1. Blu Liquid flavours and nicotine strengths.

Blu Liquid Flavour	Available in nicotine strengths
Tobacco	0.8% and 1.6%
Menthol	1.6%
Blueberry	0% and 1.6%
Cherry	0% and 0.8%
Strawberry Mint	0% and 0.8%

Figure 2. Packaging of Blu PRO Liquid flavours

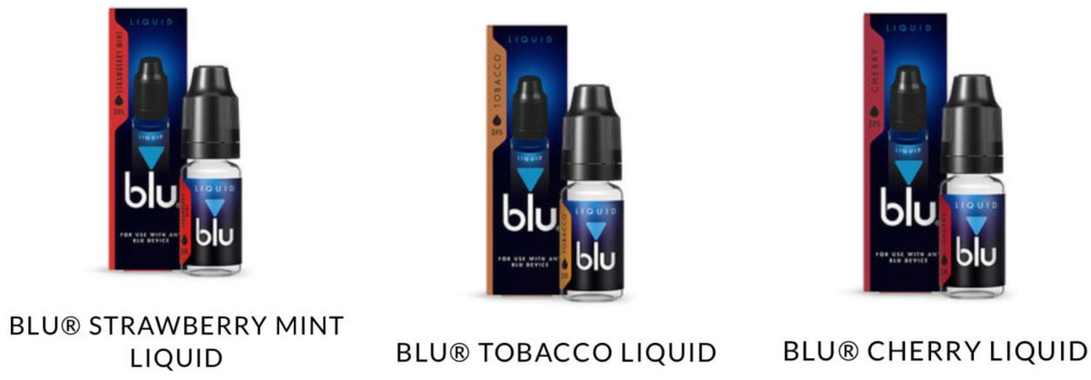

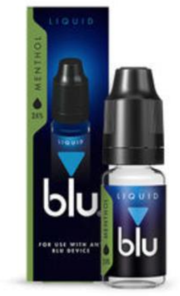

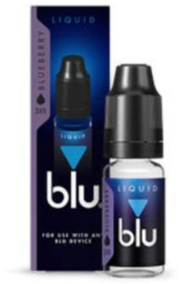

BLU® MENTHOL LIQUID BLU® BLUEBERRY LIQUID

10.1 Understanding nicotine strengths

E-liquids containing 0% nicotine means the e-liquid does not contain nicotine.

E-liquids containing 0.8% nicotine means the e-liquid contain 8 milligrams of nicotine per millilitre (i.e. 8 mg/ml).

E-liquids containing 1.6% nicotine means the e-liquid contain 16 milligrams of nicotine per millilitre (i.e. 16 mg/ml).

11 What happens if I choose to participate?

If you choose to participate in this study, you will be provided with a Blu PRO e-cigarette and three 10ml refill bottles of Blu Liquid in the flavours and nicotine strength of your choice, and given training on how to use these products.

12 What happens next?

12.1 Learning how to use the Blu PRO e-cigarette

A Study Investigator will give you training on how to assemble, charge, fill and puff the Blu PRO e-cigarette. This training will be delivered by a Study Investigator according to the *Blu Product Information Handbook*, a 15-page document that you will be given and free to take home with you.

The purpose of this training session is to ensure you can demonstrate that you are competent, confident and sufficiently knowledgeable to use the Blu PRO e-cigarette and Blu Liquids safely and effectively. In this session, the investigator will show you how to assemble, charge and refill your Blu PRO e-cigarette, and ask you to practice assembling, charging, refilling and puffing the Blu PRO e-cigarette under the Study Investigator's supervision. The investigator will also talk you through a list of health warnings associated with the use of the Blu PRO, and describe the actions that should be taken in the event of a technical malfunction or adverse health effect arising from use of the Blu PRO e-cigarette. Throughout this session, you will be encouraged to ask questions about the operation and proper use of the Blu PRO e-cigarette.

The Study Investigator will not proceed to the next stage until you feel confident in your ability to assemble, charge, refill and puff the Blu PRO e-cigarette on your own.

12.2 Vaping the Blu PRO for the first time

Following your Blu PRO training session, you will be given 30 minutes to assemble, fill and use your Blu PRO e-cigarette for the first time, and to familiarise yourself with the act and effects of puffing the Blu PRO e-cigarette. First, you will be asked, and assisted if necessary, to unpack, assemble, and fill your Blu PRO e-cigarette with a Blu Liquid of your choice. You will then be allowed to puff the Blu PRO e-cigarette as you wish for five minutes. During this time, you should try puffing the e-cigarette in different ways to find a puffing style gives you the most satisfying draw of vapour. After completing this 5-minute puffing session, you will be asked some questions about how you felt while puffing and after puffing the Blu PRO e-cigarette.

12.3 Finding your favourite flavour and nicotine strength of Blu Liquid

You will then be invited to sample any of nine combinations of flavour and nicotine strength of Blu Liquid you wish to sample. The nine combinations of flavours and nicotine strength will be contained in nine separate Blu PRO e-cigarettes, each with disposable mouth tips. You will be given a new mouth tip for each e-cigarette you wish to sample, and you are free to sample and re-sample as many combination you wish for 30 minutes. The purpose of this session if for you to find your favourite combination(s) of flavour and nicotine strength – the Blu Liquid you

can imagine you would enjoy using in place of smoking cigarettes. When you have made your choice, you will be given three 10ml bottles of your favourite liquid(s) to begin the study, but you are free to change flavour or nicotine strength of Blu Liquid at any time and as many times as you wish for the duration of your participation in the study.

12.4 Using the Blu PRO and purchasing Blu Liquid refills between day-1 and day-90

Once you have selected your favourite Blu Liquid(s), you will be provided with one Blu PRO Kit and three 10ml refill bottles of Blu e-liquid in their preferred combination(s) of flavour and nicotine strength, and then you will leave the Centre. Over the next 90 days, you are free to use your Blu PRO as often or as infrequently as you wish, free to use the Blu PRO e-cigarette at any time of the day as you wishes, and free to vape as much or as little Blu Liquid as you wishes. The only instruction we ask you to follow is to <u>try to use your Blu PRO e-cigarette instead of smoking regular cigarettes whenever you have a craving for nicotine or an urge to smoke.</u>

Once you have used your free supply of three 10ml refill bottles, you will be required to purchase, with their yown money, Blu Liquid refills from stockists in the community (e.g. Tesco, Sainsburys, Asda, and a variety of petrol stations) or from online vendors. We are asking you to buy your own e-liquid refills as and when you wish from local stockists because this replicates how e-cigarette users buy and use e-liquid refills in the real world.

<u>You will, however, be reimbursed for your purchases of Blu Liquids up to the value of £30 per month per participant, upon presentation of valid receipts.</u> A 10ml bottle of Blu Liquid in the United Kingdom typically retails for £4.99, which means you will be reimbursed for a maximum of six bottles of Blu Liquid per month. In the event that your Blu PRO e-cigarette breaks or stops working for any reason, a replacement Blu PRO e-cigarette will either be provided to you for free upon return of the broken Blu PRO e-cigarette, or you may purchase a new Blu PRO from a shop and we will reimburse you for this purchase upon presentation of a receipt and return of the broken Blu PRO. You will also be reimbursed for purchases of a new Blu PRO micro-USB charging cable in the unlikely event that the charging cable provided as part of the Blu PRO Kit stops functioning.

Reimbursement payments for Blu Liquid purchases up to the value of £30 will be made to you every 30 days by means of a personal cheque mailed to your home address or a cash payment which must be collected in person. Reimbursement payments will be made upon submission of valid paper or electronic receipts, or upon submission of electronically scanned and submitted images of receipts. Reimbursement payments will only be authorised for purchases of Blu Liquids, and only up to the value of £30 per month. It is important to emphasise that you are not prohibited from purchasing and/or using other e-cigarette brands and e-liquid brands during this study, but you will not be reimbursed for any purchases of non-Blu e-cigarette or e-liquid products.

Additionally, you must not refill the Blu PRO e-cigarette with e-liquids that are not made by Blu, and likewise you must not charge the Blu PRO e-cigarette with any charging cable other than the cable provided as part of the Blu PRO Kit.

13 Completing online questionnaires on day-30, day-60 and day-90

A requirement of participation in this study is that you complete a short questionnaire on three occasions – on day-30, day-60 and day-90. On these days, you will receive an email containing a web-link to an online questionnaire. You must click this link and complete the questionnaire within three days of receipt of this email. You can complete these questionnaires on any device (e.g. laptop, iPhone, tablet) at any time of day. Each questionnaire will take no longer than 10 minutes to complete, and will ask you questions about your experiences of using the Blu PRO in the past 30 days, and any cigarettes you have smoked.

As the completion of all three questionnaires is a requirement of your participation in this study, you will only receive a £50 stipend if you complete all three questionnaires.

14 What are the possible risks of taking part?

14.1 Risk of harm from e-cigarette use

E-cigarettes are not harmless or risk-free, which is why non-smokers are discouraged from using them. However, e-cigarette use is believed to carry a fraction of the risk associated with cigarette smoking. According to Public Health England and the Royal College of Physicians, no significant short-term health risks of e-cigarette use have been identified, and regular long-term use of an e-cigarette is believed to reduce the risk of developing a smoking-related disease by at least 95%. These health authorities therefore believe that switching completely from smoking cigarette to using e-cigarettes has the potential to generate significant health gains for the individual. The risks to bystanders arising from second-hand inhalation of e-cigarette vapour have also been found to be negligible.

The Study Investigators therefore believe it is highly unlikely that any smoker's health will deteriorate in the short, medium or long-term by switching to the Blu PRO e-cigarette, or any other e-cigarette. Indeed, the available evidence suggests a smoker’s health is likely to improve by switching to an e-cigarette. The evidence supporting these beliefs are extensively documented in the two sources below, both of which are free to download on the internet.

Public Health England. E-cigarettes: an evidence update. 2015. Accessible at: https://www.gov.uk/government/uploads/system/uploads/attachment_data/file/457102/Ecigarettes_an_evidence_update_A_report_commissioned_by_Public_Health_England_FINAL.pdf

Royal College of Physicians. Nicotine without smoke: Tobacco harm reduction. London: RCP, 2016.

14.2 Risks associated with nicotine use

The Blu PRO e-cigarette is to be refilled with Blu Liquid, which may contain nicotine, which is a highly addictive substance. However, the nicotine found in e-liquid has been drawn from the tobacco leaf, and so as you are currently a long-term cigarette smoker, you are already used to consuming a far higher quantity of nicotine through cigarette smoke than is carried by e-

cigarette vapour. Therefore, as a regular consumer of nicotine through cigarettes, we do not envisage any risks to you inhaling nicotine through e-cigarette vapour.

14.3 Risks associated with using the Blu PRO e-cigarette and e-liquids:

There is no evidence that short-term use of Blu products causes any appreciable harm to users or bystanders, where harm is characterised as any signs, symptoms or exacerbations of a health problem, acute adverse effects, pain, discomfort, or physical or psychological distress.

The Blu products that will be provided to you in this study are licensed for sale in the United Kingdom as general consumer products for purchase by individuals aged 18 years and older. Major stockists of these products include Tesco, Sainsburys and Asda supermarkets, as well as many local convenience stores and petrol stations. Blu products are not licensed or marketed as medical devices or medical products. Blu products do not claim to be effective for smoking cessation, nor have they been tested as such, nor have the long-term effects of Blu products been tested. Blu products are marketed as an alternative to smoking tobacco, for use by persons aged 18 years and older, and are not intended to be used by non-smokers.

15 What are the potential benefits of taking part?

A 2015 report commissioned by Public Health England concluded that using an e-cigarette is likely to be at least 95% less harmful than smoking a regular cigarette, and that e-cigarettes can be effective in helping smokers to quit, especially when the smoker is also provided with behavioural support. The report concluded that switching from smoking to 'vaping' (using an e-cigarette) can therefore prevent or avoid almost all the harm associated with tobacco smoking. These conclusions have been endorsed by Cancer Research UK, the Royal College of Physicians, the British Lung Foundation and many other UK health organisations. Following from this report, these organisations have adopted as policy the position that smokers who cannot or do not want to quit using nicotine should be encouraged and supported to switch from smoking cigarettes to using e-cigarettes in order to substantially reduce their risk for developing smoking-related diseases.

E-cigarette use for smoking abstinence

As a participant in this study, you will be encouraged to switch to using the Blu PRO e-cigarette for the next 90 days. As a result, you may experience several benefits from participating in this study.

One potential benefit of substituting an e-cigarette for regular cigarettes is that you may find that inhaling e-cigarette vapour reduces your nicotine cravings, and so you may have less and less of an urge to smoke. Consequently, you may find that using an e-cigarettes helps you to stop smoking cigarettes altogether, or helps you to cut down the number of cigarettes you smoke each day.

If you completely substitute e-cigarettes for regular cigarette, you may notice several positive changes in your health, in particular, your respiratory and cardiovascular health. Among the many changes that people report from as early as their first week of switching from smoking to vaping include being able to breathe more easily, coughing less frequently and less harshly, feeling less breathless, sleeping for longer without waking, coughing up less phlegm, and feeling more energetic. These short-term changes associated with switching to vaping may be a stepping stone a longer-term potential reduction in harm to your body's vital organs, and in turn, potential reduction in your risk for developing smoking-related diseases. We must emphasise though that, while there is good evidence to suggest that e-cigarettes substantially reduce exposure of your body's vital organs to harmful toxicants found in tobacco smoke, there is not yet enough evidence to say with confidence that individuals who switch from smoking to vaping will have a substantially reduced risk of developing a smoking-related disease in the future. More immediately, it must be emphasised that the positive short-term and long-term changes in health noted above vary with each individual and are not guaranteed to any individual who switches from smoking to vaping.

Another potential benefit that you may notice when you start using the Blu PRO e-cigarette is that, unlike cigarette smoke, e-cigarette vapour does not stick to your clothes or fabrics in your home, and so the smell of vapour dissipates very quickly compared to smoke, which can linger in a room for hours after the cigarette has been smoked. Many e-cigarette users also say they much prefer the fruity and sweet smells of e-cigarette vapour over the smell of tobacco smoke. If you do switch to e-cigarettes, you may find that your sense of smell and taste for food and drinks are gradually enhanced, and you may notice that the smell of stale cigarette smoke begins to leave your house, car or wherever else you would normally smoke.

16 Will my identity and data be kept confidential?

Yes. Your personal information – name, address, telephone number – will be used by the Study Investigators for the sole purpose of maintaining contact with you for the duration of your involvement in the study. Your personal information will not be analysed, reported or shared in any way. Your personal information will be stored in paper form only and stored in a key-locked metal filing cabinet in the offices of the Centre for Substance Use Research. Only the Study Investigators will have key access to this cabinet. The Study Investigators will be prohibited from taking any documents that contain your personal information off the premises of the Centre for Substance Use Research. Your contact telephone number will also be stored on a password-protected study cell phone (an iPhone 6S). This phone will be used to allow you to contact the Study Investigators out of normal office hours. Only the Study Investigators will have password access to their phone. At the conclusion of your involvement in the study, all of your personal information will be securely destroyed.

The answers you give in response to the online questionnaires will be stored securely in a password-protected online database. This database will not be accessible to or shared with any individual other than the Study Investigators. Upon acceptance into the study, you will be assigned a unique participant number; only the unique participant number will be stored and linked to your questionnaire responses. The online questionnaires ask you to provide any further personal information, and it will not be possible for any person to deduce your identity on the basis of your responses to any question in any questionnaire. After the unique participant number has been assigned, no person other than the Study Investigators will be able to match your personal information to your questionnaire responses.

Confidentiality of your electronically-submitted data will be assured to the degree permitted by the technology being used. All computers belonging to the Centre of Substance Use Research are protected against malware attacks and other attempted intrusions by anti-virus software, *BitDefender*. All possible steps have been taken to protect your privacy.

17 Compensation for taking part and reimbursements

You will be reimbursed for all travel expenses incurred as a result of participating in this study, including the costs of your travel to the Centre today, upon presentation of receipts.

You will also be reimbursed for purchases of Blu Liquid refills up to the value of £30 per month upon presentation of store receipts.

We will also pay you £50 for completing the online questionnaires.

18 Who funded this research study?

Fontem Ventures, a company that makes e-cigarettes, and a wholly owned subsidiary of Imperial Brands PLC (formerly Imperial Tobacco) provided funding for this study. Although *Fontem Ventures* provided funding for this study, it is not involved in any way with the running of this study.

19 What if I change my mind?

Your participation in this study is entirely voluntary. You are free to withdraw from this study at any stage without giving a reason. You are also free to go home and think about whether you wish to take part, to discuss your decision with friends and family, and contact the Study Investigator when you have come to a decision.

20 How can I find out more about this study?

If you have any questions about any aspect of this research study, or any questions about what would be required from you, please ask the Study Investigator, or feel free to call or email any of the study investigators using the details provided above.

21 I’ve decided I want to take part. What happens next?

If, after reading all this information carefully, you decide that you wish to participate in this study, the Study Investigator will ask you to read and sign an Informed Consent Form, which the Study Investigator will co-sign and date. You will then be ready to start the study.

22 I’ve decided I don’t want to take part. What happens next?

If you have decided that do not wish to take part in this study, the Study Investigator will conclude your interview and reimburse you for your travel expenses incurred by coming to the Centre for Substance Use Research today. On behalf of the Centre, we thank you for coming here today and we wish you all the best for the future.

END
