# Supplementary material for: The Value of Providing Smokers with Free E-Cigarettes: Smoking Reduction and Cessation Associated with the Three-Month Provision to Smokers of a Refillable Tank-Style E-Cigarette
Source: Int J Environ Res Public Health. 2018 Sep 3;15(9):1914. doi: 10.3390/ijerph15091914 (PMC6165311; doi:10.3390/ijerph15091914)
Supplement: Supplementary file 1 [file ijerph-15-01914-s001.zip › Supplementary File 2. Blu PRO Kit Information Book.pdf]

Blu PRO Kit

Information Booklet

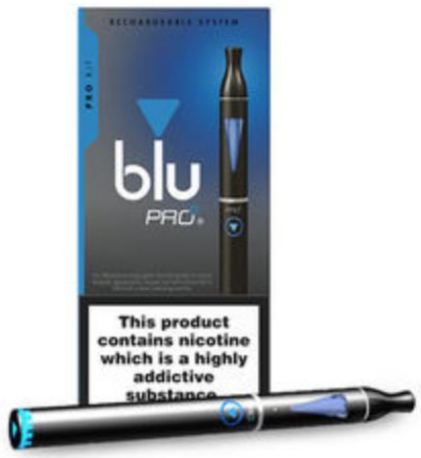

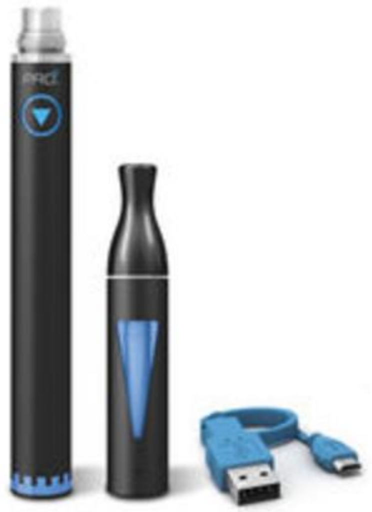

This Blu PRO Kit Information Booklet is solely for use by participants of the following study:

"The effect of using the Blu PRO e-cigarette in place of smoking conventional cigarettes for 90 days,"

Table of Contents

1 What's in the Blu PRO Kit? 3

1.1 E-liquid flavours and levels of nicotine 3

1.2 Understanding nicotine strengths..5

2 How do I charge my Blu PRO® Kit? 6

2.1 Charging warning...6

3 How do I know when I need to charge my Blu PRO? 7

4 Filling your clearomiser 8

5 Using your Blu PRO® 10

6 Reporting technical problems with your Blu PRO Kit or Blu Liquids 12

7 Warnings...13

8 Recycling and disposal...15

1 What's in the Blu PRO Kit?

Welcome to your guide on how to use your Blu PRO Kit. In this booklet, you'll find everything you need to understand how to use your Blu PRO e-cigarette safely and effectively.

The Blu PRO Kit (shown below in Figure 1) contains:

- 1 x Blu PRO rechargeable device (battery 1100mAH +/-7%)
- 1 x Blu PRO clearomiser
- 1 x Blu PRO USB Charger

Figure 1. The Blu PRO Kit.

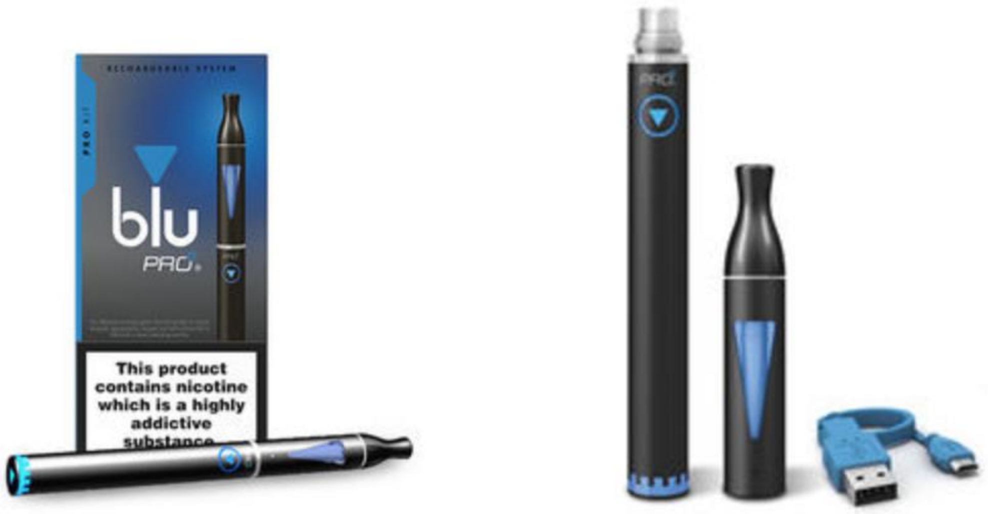

1.1 E-liquid flavours and levels of nicotine

The Blu PRO is an open-system electronic cigarette, which means it is to be refilled with e-liquid. The Blu PRO e-cigarette is to be refilled with Blu Liquid. Blu Liquids are packaged in 10ml plastic bottles, and contain vegetable glycerine, propylene glycol, flavourings, nicotine and water. Blu Liquids are available for retail purchase in the United Kingdom, to individuals aged 18 years and older, in nine combinations of five flavours and three nicotine

strengths, shown in Table 1 below. Images of the packaging of the five flavours of Blu Liquid are shown in Figure 2 below.

Table 1. Blu Liquid flavours and nicotine strengths.

Blu Liquid Flavour	Available in nicotine strengths
Tobacco	0.8% and 1.6%
Menthol	1.6%
Blueberry	0% and 1.6%
Cherry	0% and 0.8%
Strawberry Mint	0% and 0.8%

Figure 2. Packaging of Blu PRO Liquid flavours

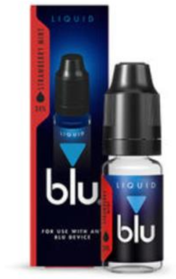

BLU® STRAWBERRY MINT LIQUID

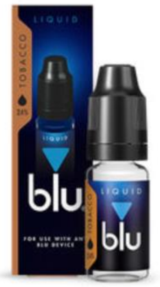

BLU® TOBACCO LIQUID

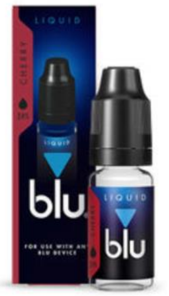

BLU® CHERRY LIQUID

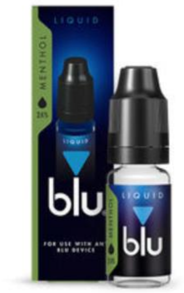

BLU® MENTHOL LIQUID

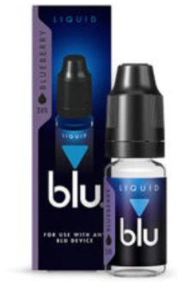

BLU® BLUEBERRY LIQUID

1.2 Understanding nicotine strengths

- E-liquids containing 0% nicotine means the e-liquid does not contain nicotine.
- E-liquids containing 0.8% nicotine means the e-liquid contain 8 milligrams of nicotine per millilitre (i.e. 8 mg/ml).
- E-liquids containing 1.6% nicotine means the e-liquid contain 16 milligrams of nicotine per millilitre (i.e. 16 mg/ml).

2 How do I charge my Blu PRO® Kit?

To start using your Blu PRO e-cigarette, you first need to charge it. Although the rechargeable device comes with a little charge, its best to charge it fully for a more satisfying vaping experience.

To charge your Blu PRO® rechargeable device, complete the following steps:

1. Screw your Blu PRO rechargeable device into the USB Charger.
2. Plug the USB charger into a USB-compatible charging port.
3. The light on the USB charger will glow red and the tip of the device will flash to indicate that it has started to charge.
4. Once the device is fully charged, the tip will blink red and turn Blue. It takes approximately three hours to fully charge the Blu PRO rechargeable device.

If the light does not glow when the USB charger is plugged in, the device has not been screwed in correctly. Simply unscrew the battery from the charger and reconnect.

2.1 Charging warning

Do not use any charging device that has not been created by Blu, and do not charge for an extended period of time. If you are not sure whether a charging device was created by Blu, do not use it to charge your Blue PRO® kit.

Do not leave your Blu PRO® connected to charging cable for longer than 24 hours.

If the charger you have been provided with fails to charge your Blu PRO® rechargeable device, or if the charger becomes damaged in any way, do not use the charger. Call Dr Christopher Russell immediately at **07955460277** to arrange for safe disposal of the defective charger and for delivery of a new compatible charger. Under no circumstances should you try to charge your Blu PRO® Kit with a defective charger, or a charger that was not created by Blu.

3 How do I know when I need to charge my Blu PRO?

When the button your Blu PRO rechargeable device flashes red, this indicates that the rechargeable device needs to be recharged. There is also an indicator on the front of the rechargeable device that displays an amount of remaining charge left in the rechargeable device.

When your Blu PRO Battery starts to run out of charge, you will start to notice a few changes in the way your Blu PRO is performing. This may include less vapour production and a less intense flavour. When you notice any of these things, this is a good time to prepare to recharge as your charge will soon be completely depleted. When your charge has depleted, the Blu PRO will stop producing vapour.

To get the best vaping experience, you should start to re-charge your Blu PRO as soon as your device starts to produce less vapour and a less intense flavour.

4 Filling your clearomiser

When your Blu PRO is charged, the next step is to fill the clearomiser with Blu Liquid. To be extra safe, you can lay down a few paper towels.

To fill your clearomiser with Blu Liquid, complete the following steps:

1. Unscrew the mouthpiece from the top of the clearomiser.
2. Open the Blu Liquid bottle by pushing down on the cap and turning it to the left.
3. Place the nozzle of the liquid bottle against the inside of the wall of the clearomiser. Be sure to avoid the centre tube of the clearomiser.
4. Squeeze the liquid bottle until the liquid reaches the top of the triangle in the clear window of the clearomiser. Do not fill past the top of the triangle.
5. Replace the lid on the Blu Liquid
6. Screw the mouthpiece back onto the clearomiser, and then screw the clearomiser onto the rechargeable device.
7. Allow a few moments for the liquid to settle.
8. Before using your Blu PRO device or activating the rechargeable device, prime your clearomiser by taking a few quick, short puffs in order to saturate the wick in liquid.

You should replace your Blu PRO clearomiser after every 10 liquid fills to ensure a consistent, quality experience.

How to use your Blu PRO Kit

Figure 3. How to fill the clearomiser and connect to the Blu PRO battery and mouth tip.

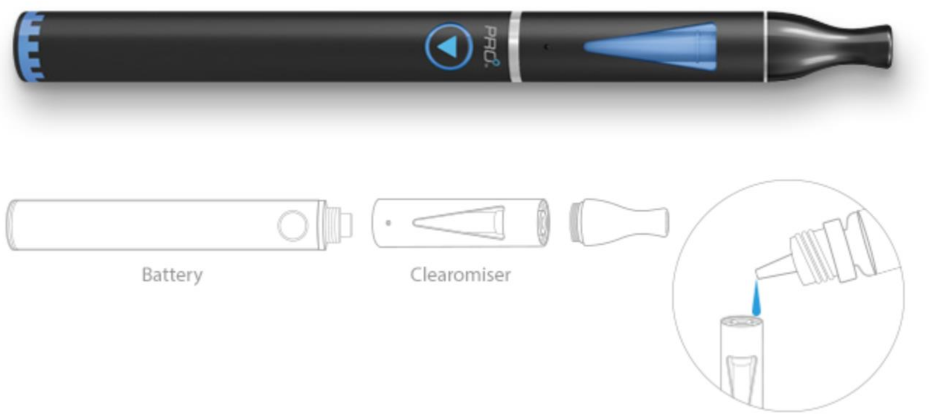

5 Using your Blu PRO

Once you have charged the rechargeable device and connected it to the filled clearomiser, you are ready to start using your Blu PRO.

To use your Blu PRO, complete the following steps:

1. Place the mouthpiece in your mouth and push the button 5 times in quick succession. The button and rechargeable device will flash 3 times to show that it is activated.
2. Push and hold the button on the Blu PRO rechargeable device every time you take a puff. The button will light as you press it. While holding the button, inhale slowly and deeply. This will vapourise the liquid, allowing you to inhale and exhale vapour.
3. Before you take your first deep draw from the mouthpiece, take a few short inhales to get the wick coated in liquid.
4. To turn the Blu PRO off, push the button 5 times in succession again. Your Blu PRO will flash 3 times to indicate that it has been turned off. You can verify that the Blu PRO is inactive by pressing the button on the rechargeable device. If the rechargeable device does not light, the device is turned off.

You should always turn off the Blu PRO when it is not in use. When the button on the Blu PRO rechargeable device flashes red, the device needs to be recharged.

If you are ever in doubt about how to use the Blu PRO, you can watch a tutorial video at the website below. Simply copy the link below into your internet browser to watch the video.

https://www.Blu.com/en/GB/explore/about-Blu-products/how-the-Blu-pro-kit-works/how-the-Blu-pro-kit-works.html

How to use your Blu PRO Kit

Figure 4. How to hold the Blu PRO.

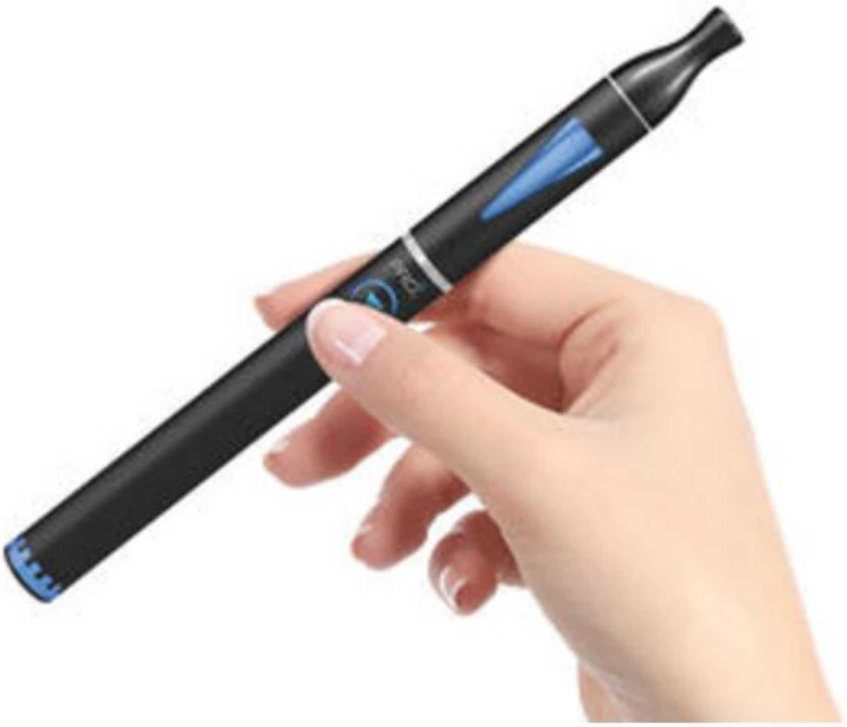

6 Reporting technical problems with your Blu PRO Kit or Blu Liquids

Before you use any Blu product, always inspect them to ensure that you do not see any signs of damage prior to each use, including:

- Cracks or deformities.
- Loose or ill-fitting parts.
- Leakage of a liquid.

Should any e-liquid be visible on the mouthpiece, wipe with paper or a dry cloth prior to use.

Do not use Blu products if the packaging has been damaged or opened.

Do not store Blu products in direct sunlight or store above 25C or below 2C. do not expose or immerse the products in water.

Do not use Blu products in high-oxygen environments, especially when using hom-oxygen therapies.

If you experience any of the following problems, stop using the Blu PRO immediately and contact a Study Investigator and, if necessary, a medical professional:

- If you experience any problems while using, charging, assembling or refilling your Blu PRO.
- If your Blu PRO breaks, snaps, fails to re-charge, over-heats, or combusts.
- If you become ill or experience any adverse health effects from using your Blu PRO.

7 Warnings

Please read the following 10 warnings carefully before using any Blu products.

1. Blu products are for use by persons aged 18 years and older, and are intended for use by adult smokers.

2. Blu products are not smoking cessation products and have not been tested as such, nor have the long-term effects of using Blu products been established.

3. Blu products contain nicotine which is a highly addictive substance. Do not use Blu products if you have a medical condition, are pregnant, are planning to become pregnant, are breastfeeding, have cardiovascular disease, have renal or hepatic impairment, have adrenal or thyroid gland disorders, have diabetes, have lung disorders, have high blood pressure or are taking medicine for depression or asthma.

4. Blu Liquids are manufactured in a facility that may use equipment that is exposed to trace amounts of tree nuts and/or peanuts.

5. If you become ill or experience any adverse health effects from using Blu products, stop using them immediately and consult your doctor. If any liquid is swallowed, call a poison centre or your doctor if you feel unwell.

6. If any liquid gets on your skin, wash with plenty of water. Wash your hands thoroughly after handling Blu Liquid.

7. Blu products may contain small parts and could present a choking hazard. Keep all Blu products out of reach of children and animals.

8. Do not use Blu products in high-oxygen environments, especially when using home-oxygen therapies.

9. Some people may experience side effects when using e-cigarettes. Immediately stop use of Blu products, seek medical advice and notify the study leader/staff immediately (see contact details below) if you experience any of the following: development of an irregular heartbeat; allergic reaction such as rash, itching or swelling of the tongue, mouth or throat; feeling faint; nausea; headache; or any other unusual or adverse effect.

10. Respect others when using Blu products as some venues and locations do not allow electronic cigarette use. Familiarize yourself with any electronic cigarette policies before using Blu products in public venues.

8 Recycling and disposal

Blu products in scope of the EU WEEE Directive are labelled with a crossed-out “wheelie bin” symbol as required by this directive. This symbol indicates that the product was placed on the market after August 13, 2005, and that users should segregate the product from other waste at end-of-life.

This symbol on Blu products or their packaging means that the product should not be disposed of with your other household waste.
